# Exploring the potential of rapid maxillary expansion and masticatory muscle activity in unilateral posterior crossbite

**DOI:** 10.4317/jced.61604

**Published:** 2024-06-01

**Authors:** Gabriel-Pereira Nunes, Maria-Juliana-Sismeiro-Dias Morabito, Larissa-Pereira Nunes, Letícia-Cabrera Capalbo, Alexandre-Henrique-dos Reis Prado, Priscila-Toninatto-Alves de Toledo, Mayra-Fernanda Ferreira, Arles-Naisa-Amaral Silva, Tamires-Passadori Martins, Natália-Helena Colombo, Túlio-Morandin Ferrisse

**Affiliations:** 1 Department of Preventive and Restorative Dentistry, School of Dentistry, São Paulo State University (UNESP), Araçatuba, São Paulo, Brazil; 2 Laboratory for Bone Metabolism and Regeneration, University of Porto, Faculty of Dental Medicine, Porto, Portugal; 3 Department of Cariology, Restorative Sciences, and Endodontics, School of Dentistry, University of Michigan, Ann Arbor, MI, United States of America; 4 Department of Restorative Dentistry, School of Dentistry, Federal University of Minas Gerais (UFMG), Brazil; 5 Oral Medicine, Department of Diagnosis and Surgery, School of Dentistry, São Paulo State University, (UNESP), Araraquara, São Paulo, Brazil; 6 Department of Preventive Dentistry, Periodontology and Cariology, University Medical Center Göttingen, Göttingen, Germany

## Abstract

**Background:**

This systematic review and meta-analysis aimed to evaluate if rapid maxillary expansion improves the activity of the masticatory muscles (masseter and temporal) in patients with unilateral posterior crossbite.

**Material and Methods:**

Searches were performed in PubMed/MEDLINE, Scopus, Web of Science, Embase, Cochrane Library, and grey literature. A manual search of orthodontic journals was also performed. Randomized clinical trials or longitudinal prospective studies were eligibles. Meta-analyses were conducted using R software with the “Meta” package, applying mean differences with a 95% confidence interval. Risk of bias was assessed using the Newcastle-Ottawa scale, and evidence certainty was evaluated using GRADE.

**Results:**

Nine articles were included. Qualitative analysis showed that RME treatment in patients with unilateral posterior crossbite showed a positive correlation with improvement in masseter and temporalis muscle activity. Meta-analyses indicated a significant difference for all models of muscle activity after treatment with rapid maxillary expansion, except for the temporal muscle in the force exerted on the maximum voluntary clenching on cotton rolls. The studies showed low bias risk, and the evidence certainty for each analysis was generally low to very low.

**Conclusions:**

This investigation demonstrated the benefits of R rapid maxillary expansion in treating unilateral posterior crossbite and its potential therapeutic effects on the masticatory muscles.

** Key words:**Rapid maxillary expansion, masticatory muscles, unilateral posterior crossbite, systematic review, meta-analysis.

## Introduction

Posterior Crossbite is a common type of malocclusion that has a high prevalence in children, affecting up to 22% of pediatric orthodontic patients in the primary and mixed dentition (1,2), and 15% of individuals in general (3), thus characterizing one of the most common orthodontic problems of the occlusal development phase (1,4). This occlusal problem is defined as an abnormal buccolingual relationship of one or more teeth in the maxilla with one or more teeth in the mandible, when the dental arches are in a centric relationship, and can be bilateral or unilateral (5), the latter being the most common, occurring in 80 to 97% of individuals with PCB (6).

Although the etiology of posterior crossbite does not have its etiology well established in the scientific literature (7), its multifactorial nature is known and may be associated with deleterious habits such as non-nutritive sucking (8-10), heredity, mouth breathing pattern, and adenoid hypertrophy (11,12), in addition to bruxism, tongue thrusting and habit of biting objects (13,14). Patients with unilateral posterior crossbite generate an asymmetrical stimulus of the masticatory muscles, as the mandible starts to make lateral movements to one side or the other to make the posterior teeth touch (15,16). This occlusal change is not corrected spontaneously if there is no early intervention after diagnosis, consequences may reflect in the patient’s adult life (17,18). In long term, there may be an overload of the jaw muscles and joints, thus contributing to skeletal asymmetries and temporomandibular joint disorders (16,17,19).

Thus, treatments to correct posterior crossbites and prevent further problems in children have been used. Among these, rapid maxillary expansion is a widely accepted intervention (20), being the most common used to correct this type of malocclusion. Opening the midpalatal suture increases the transverse width of the maxilla (21) and the dental arch perimeter, allowing the correction of maxillary constriction related to posterior crossbite (20). Regardless of the type of palatal expander used, rapid maxillary expansion is an effective procedure capable of producing skeletal effects in the maxilla (21).

In addition, muscle imbalance during rest, speech, chewing, and swallowing can cause favorable conditions for the development, maintenance, or recurrence of malocclusion (22). Therefore, orthodontic treatment should include not only the correction of malocclusion but also the restoration of altered stomatognathic functions present in this type of malocclusion (23-24). Whereas a previous systematic review aimed to assess whether, in children treated orthodontically for unilateral posterior crossbite, it showed that functional asymmetry improves after treatment (25), however, it was reported that the results should be interpreted with caution due to the number of studies identified being very small, where only four included studies were evaluated by electromyography so that two of these studies presented controversial results regarding the measured muscle activities. Therefore, this study has limitations, and a complete assessment of the subject is needed. In addition, new clinical trials have been published, which can increase the robustness of a systematic review, with a more adequate sample size and possibly conducive to finding more homogeneous outcomes.

Thus, the assessment of muscle activity during mastication and jaw rest, both in unilateral posterior crossbite e and after its correction, will significantly contribute to myofunctional and orthodontic therapy, as muscle activity can interfere with the stabilization of occlusal correction (8,15,26). Given this presumption and the limited systematic evidence regarding the impact of orthodontic treatment using RME on the activity of masticatory muscles, this systematic review and meta-analysis aimed to assess whether such treatment enhances the activity of masticatory muscles, specifically the masseter and temporal muscles, in individuals with unilateral dental crossbite.

## Material and Methods

-Protocol and registry

This systematic review was registered in the International Prospective Registry of Systematic Reviews (PROSPERO - CRD42021245264) and structured according to the Checklist of Preferred Reporting Items for Systematic Reviews and Meta-Analysis (PRISMA) (27), by the guidelines in the Cochrane Handbook (28), and recently published systematic reviews (29-31).

-Eligibility criteria and question PICO

Inclusion criteria were: i) randomized clinical trials or longitudinal prospective studies evaluating functional outcomes both before and after the treatment of functional unilateral posterior crossbite in growing children; ii) children with primary or mixed dentition; iii) Studies from which the outcome of interest was functional measurement (masseter and temporal muscles), by electromyography, before and after the functional treatment of posterior crossbite. The exclusion criteria consisted of: i) studies in which the investigators did not provide data related to intervention and comparison groups; ii) studies with less than ten children; iii) studies with adults, as well as studies including patients with cleft lip and palate, craniofacial syndromes, or medically compromised patients; iv) retrospective studies, cross-sectional studies, case series, case reports, non-human studies, literature reviews, and studies based on expert opinions. No restrictions were imposed to language and publication period.

The population, intervention, comparison, outcome (PICO) approach was used to address the following question: “Does rapid maxillary expansion improve the activity of the masticatory muscles in patients with posterior crossbite?”. The study population was patients with a posterior crossbite. The intervention was treatment with rapid maxillary expansion, and the comparison was data from the baseline or control group. The outcome was electromyographic activity of temporal and masseter.

-Sources of information and search strategy

Two independent authors (MJSDM and GPN) conducted an electronic search in the following electronic databases: PubMed/MEDLINE, Scopus, Web of Science, Embase, and Cochrane Library. A specialized librarian guided the entire electronic search strategy. A manual search was also performed to identify manuscripts that the electronic search might not have retrieved. To find unpublished or ongoing studies, the registry of clinical trials was investigated on the website ClinicalTrials.gov (www.clinicaltrials.gov), without restriction on the date or language of publication.

The search was carried out until March 03rd, 2024. A specialized librarian guided the entire electronic search strategy, and it was performed with MeSH terms/entry terms and free terms appropriately adapted for the databases (Supplement 1) (http://www.medicinaoral.com/medoralfree01/aop/jced_61604_s01.pdf). A manual search in area-specific journals was carried out to complement this review, including the following journals: European Journal of Orthodontics, Progress in Orthodontics, American Journal of Orthodontics and Dentofacial Orthopedics, The Angle Orthodontist, Orthodontics & Craniofacial Research and Journal of Electromyography and Kinesiology. In addition, the grey literature (produced at governmental, academic, entrepreneurial, and industrial levels, in printed or electronic format, yet not controlled by commercial publishers) was examined using the OpenGrey database (http://www.opengrey.eu/http:// www.opengrey.eu/).

-Study selection and data extraction process

Initially, the articles were selected by the title and abstract according to the pre-established eligibility criteria. All discrepancies were analyzed by a third reviewer (TMF) through a consensus meeting. One of the authors (GPN) collected the relevant information from the articles, and a second author (MJSDM) reviewed it. The following variables were collected from the articles: author/year (local), study design, sample size (n) and type of posterior crossbite, mean age at the start of treatment in years, mean duration of therapy in months, outcome variable, outcomes evaluated, follow-up, outcomes: muscle activity of the temporal and masseter, and conclusion. The kappa score was applied to calculate the interexaminer agreement during the inclusion process for publication-evaluated databases. Any disagreements were resolved by discussion and consensus of all authors.

-Quality Assessment and Risk of Bias

The Newcastle-Ottawa scale qualifier was used to assess the risk bias of the selected non-RCT studies (prospective studies). The Newcastle-Ottawa scale is based on three major components: selection, comparability, and outcome for cohort studies. According to that quality scale, a maximum of 9 stars can be given to a study, representing the highest quality. Five or fewer stars represent a high risk of bias, whereas six or more stars represent a low risk. Then, the selection can provide four stars, two stars can be allotted to the compatibility, and three stars can be given for the exposure (32).

-Summary measurements

The quantitative analyses were performed using R software with the “Meta” package, version 3.6.3, to evaluate the effect on muscle activity (Masseter and Temporal muscles) before and after the treatment of rapid maxillary expansion. Eight studies were included in the meta-analysis, and the sub-groups were formed according to variable measurement tools (AMR, AMR TENS, COTTON, and CLENCH) and by the muscles (Masseter and Temporal muscles). The mean difference (MD) was the effect measure required, and the random effect model was applied with a 95% confidence interval (CI). Heterogeneity was tested using the I2 index, which was considered substantial or high to the I2 index ≥ 50%. The funnel plot (n=2) and the trim-and-fill method (n ≥3) were used to assess the publication bias. In addition, the trim-and-fill method was also used to evaluate bias in meta-analysis (33).

-Certainty of the evidence

The quality of the evidence (certainty in the estimates of effect) was evaluated using the Grading of Recommendations, Assessments, Development, and Evaluations approach (GRADE) criteria using the software GRADEpro GDT. The included articles were assessed according to study design, risk of bias, inconsistency, indirectness, and imprecision (34). Additionally, the quality of the evidence may be upgraded if the magnitude of the effect is either large or very large or if all plausible confounding factors reduced the effect or indicate the presence of a spurious effect. Therefore, the quality of the evidence can vary from very low to high. The evaluations were carried out by two researchers independently (GPN and MJSDM) and then compared.

## Results

-Study selection

**Figure 1:**
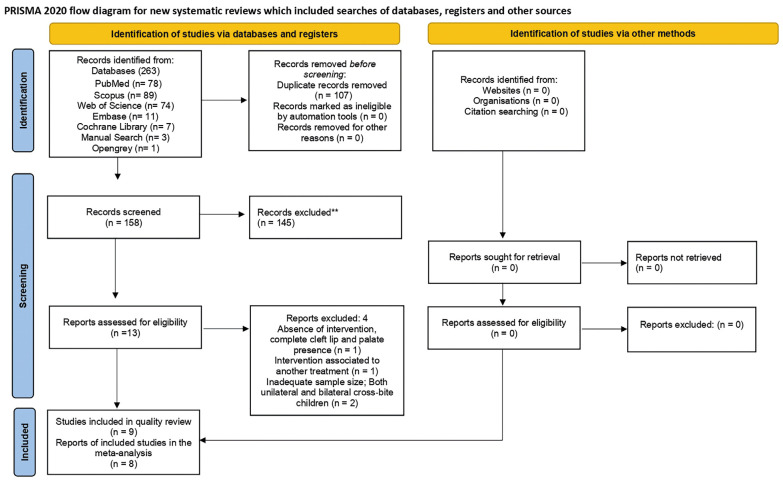
Flow diagram of search in databases according to PRISMA 2020 Statement.

The database search retrieved 263 studies: 78 from PubMed/MEDLINE, 89 from Scopus, 74 from Web of Science, 11 from Embase, seven from Cochrane Library, three from manual search, and one from Open Grey (Fig. 1). After duplicates were removed, a total of 158 studies remained for the evaluation of titles and abstracts. Subsequently, 13 articles were selected for full reading, with four articles excluded after assessing the eligibility criteria (8,35-37) (Supplement 2) (http://www.medicinaoral.com/medoralfree01/aop/jced_61604_s02.pdf)). Thus, nine prospective studies were included in the qualitative analysis (18,20,38-44). The kappa score for articles included in all databases showed an almost perfect level of interexaminer agreement (k = 0.92).

-Characteristics of the studies 

The characteristics of the nine studies are listed in  Table 1. A total of 298 patients with unilateral posterior crossbite, with a mean age of 9.4 years. The included studies were developed in four different countries: Italy (18,39,41-43), Spain (40), Brazil (20;44), and Turkey (38). All included studies evaluated patients without systemic diseases. The mean duration of interventions in the included studies varied between two to six weeks (39,43), three months (38), or until posterior crossbite overcorrection (18,20,40-42,44). The muscle activity was verified in the following situations: chewing (20,39,40,44), rest position (AMR) (18,20,38,40,41,44), rest after transcutaneous electrical neural stimulation (AMR TENS) (18,40), maximum voluntary clenching on teeth (CLENCH) (18,20,38,40-44), maximum voluntary clenching on cotton rolls (COTTON) (18,41), and during the swallowing (38,40).

-Quality assessment and risk of bias of included studies 

The risk of bias was analyzed through the Newcastle-Ottawa scale ( Table 2). All included studies were classified with a low risk of bias (18,20,38-43). However, minor pitfalls were identified among articles, such as those related to the sample selection (18,20,40-42) and comparability (40-43) ( Table 3). Besides the standardized methods, the absence of systemic diseases and the use of statistical methods to reduce the confounding factors may reduce the risk of bias in the included studies.

-Meta-analysis and Certainty of Evidence

Three studies were included in the meta-analysis (18,20,41). Sub-groups were formed according to the methods section. There was only non-statistical significance for CLENCH and AMR right side for the masseter muscle and CLENCH and COTTON right and left side related to the temporal muscle. In most of the results for both muscles, there was no identification of the publication biases according to funnel plot analysis. The heterogeneity of the meta-analysis results has shown a significant variation (0%-98.7%). However, more reliable results are those that showed I2 < 50%. In short, the treatment of RME has significant results in the activity of the masseter and temporal muscles ( Table 3). For masseter muscle only for CLENCH’s right side and COTTON’s right side, there was a significant reduction of muscle activity after the treatment of RME. In contrast, there was a significant increase in muscle activity for the temporal muscle only for AMR on the right side and AMR TENS on both sides.

**Figure 2:**
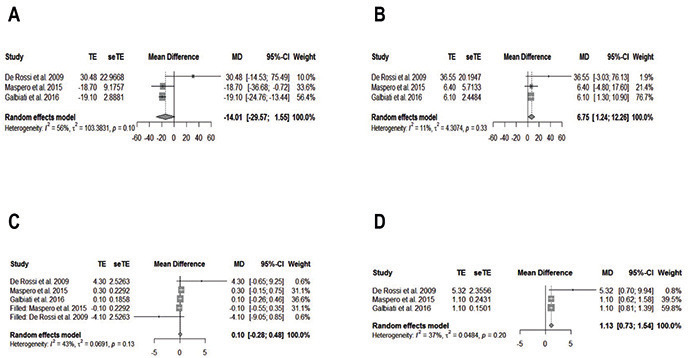
Forest plot and the trim-and-fill method for masseter muscle. A. AMR on the right side. B. AMR on the left side. C. CLENCH on the right side D. CLENCH on the left side. SD = standard deviation; MD = mean difference; CI = confidence interval; TE = estimated mean; SeTE = estimated standard deviation.

**Figure 3:**
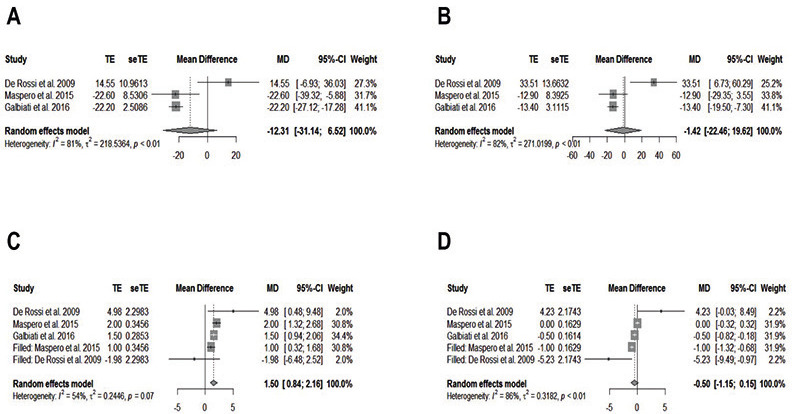
Forest plot and the trim-and-fill method for temporal muscle. A. CLENCH on the right side. B. CLENCH on the left side. C. AMR on the right side. D. AMR on the left side. SD = standard deviation; MD = mean difference; CI = confidence interval; TE = estimated mean; SeTE = estimated standard deviation.

For n = 3 was performed an additional analysis was named the trim-and-fill method. There were significant results for masseter muscle in AMR on the left side and CLENCH on the left side (Fig. 2A,B,D). There were only publication and meta-analysis biases for AMR on the right side (Fig. 2C). On the other hand, there was only statistical significance in temporal muscle for AMR on the right side (Fig. 3A-C), and publication and meta-analysis biases were identified for AMR on the right and left sides (Fig. 3C,D). The GRADE approach was used in all meta-analysis groups and subgroups and presented “low” and “very low” levels of evidence ( Table 3).

## Discussion

As previously mentioned, unilateral posterior crossbite is one of the most frequent malocclusions in children and does not present a tendency towards spontaneous correction. For this reason, after the orthodontic diagnosis, it is highly recommended that the treatment with maxillary expansion starts as early as possible. Deep knowledge of biomechanics and muscular characteristics is essential when planning orthodontic treatment. Understanding the correlation between masticatory muscles with skeletal malocclusion and craniofacial morphology may magnify craniofacial orthopedics’ results that ensure treatment stability (45).

The results of the present systematic review indicated a positive correlation in the improvement of electromyographic activity in patients with unilateral crossbite after treatment with rapid maxillary expansion. The nine articles included in the present study showed that after rapid maxillary expansion, there was a significant difference in all muscle activity models evaluated (CLENCH, AMR, AMRTENS, COTTON, SWA), except for the temporal muscle in COTTON. The fact that this difference was not observed is mainly due to the anatomical location of the temporal muscle, which does not undergo variations during the activity of the cotton model, as it is a posterior region, which ends up demanding this muscle during clenching (18,20,38-44).

In this study, an electromyography approach was standardized and used. This method is considered objective and reliable in the analysis of variations in the electrical system of masticatory muscles because, through the standardization of electromyographic signals and normalization of the data and outcome variables evaluated, it reduces the biological bias, allows the analysis between groups and intragroup in a more accurate, appearing in a protocol widely used and validated in many scientific studies in observed analysis periods (baseline and post-treatment) (39,43). Furthermore, it can obtain information from muscle conditions on dynamic and static contractions (39).

Rapid maxillary expansion is a method for the treatment of the maxillary complex and is often used in patients with unilateral crossbites (18,28). Moreover, using appliances such as Quad-Helix, Haas, and Hyrax is proven effective and faster than removable appliances for slow maxillary expansion (46). Therefore, it is extremely important to evaluate the effects of interceptive treatment with rapid maxillary expansion through electromyography to confirm the neuromuscular system’s adaptation and the patient’s current occlusal condition after treatment (41).

Maspero *et al*. (18) observed that rapid maxillary expansion led to increased muscle activity in the masticatory muscles during chewing, along with significant changes in patients’ muscle tone. Additionally, electromyography evaluations revealed a noTable increase in muscle activities of the masseter and temporalis muscles after the removal of the expander device, accompanied by enhanced maximum mouth opening. These findings are consistent with those reported by Galbiati *et al*. (41). Similarly, Spolaor *et al*. (39) found that patients undergoing rapid maxillary expansion treatment experienced improvements not only in dental and skeletal structures but also in nasal airways and functional activities of masticatory muscles.

A recent systematic review observed that patients with posterior crossbite exhibit alterations in the electromyographic activity of the masticatory muscles, and in cases of unilateral crossbite, patients demonstrate asymmetric electromyographic activity when the side of the crossbite is compared with the side without crossbite (47). These findings reinforce the importance of rapid maxillary expansion treatment for improving masticatory muscle activity, as elucidated in the present systematic review. It is known that functionally, changes in masticatory musculature are associated with variations in muscle strength. Meanwhile, from an electromyographic perspective, these changes are associated with a lower or higher number of recruited motor units in the time domain.

The studies indicated that following crossbite correction, there was an increase in muscle activity observed during deliberate chewing on both the right and left sides, with a significant difference noted. In other words, electromyography demonstrated that rapid maxillary expansion facilitated muscle balance on both sides, leading to improved jaw positioning and enabling symmetrical growth, particularly during the active growth phase of patients (48). Similar findings were observed during chewing on the non-crossed side (44). Occlusal disturbances lead to a reduction in masticatory strength, particularly on the side affected by the crossbite, and they also impact the activity of the muscles during contraction. These disturbances result in a smaller surface area for chewing, which consequently reduces both the duration and strength of mastication (49). The action potential generated during chewing contractions is influenced by factors such as the surface area available for chewing, the duration of mastication, and the texture of the food being consumed (50). The reduction in chewing area is typically observed in molars and premolars affected by the crossbite, as well as in the positioning of molars that do not occlude normally. Electromyographic monitoring reveals frequencies with both high and low amplitudes on the side with the crossbite, whereas on the non-crossbite side, there are several amplitude peaks that gradually increase in magnitude but become more attenuated as they approach the calibration limit (51).

Early diagnosis of posterior crossbite in children and adolescents is crucial for proper development of the stomatognathic system, including muscular function (43,52-54). Recent studies emphasize its significance in preventing long-term complications and guiding timely interventions for optimal oral health outcomes (43,52). In a prior investigation (43), an examination of electromyographic symmetry indices among adolescents with and without crossbite demonstrated that muscle activity imbalance, although more pronounced than in healthy adults, was not directly associated with unilateral crossbite during both stationary and moving tasks. Nonetheless, the impact of this malocclusion on asymmetry indices has been noted in adults with unilateral crossbite (55). It is conceivable that this relationship is less discernible during childhood and adolescence due to the presence of other factors, such as dental development and dietary habits. Consequently, a comparison of activity levels between the affected and unaffected sides facilitated a more detailed assessment, revealing reduced activity disparities following RME in all assessments, implying that the proposed treatment fostered equilibrium and development of the stomatognathic system. Extending the observation period for these patients might unveil more substantial alterations requiring longer durations to materialize. Nevertheless, the of at least three-month adopted in the eligible studies sufficed to detect alterations favoring the overall morphophysiological system under consideration.

Although the prospective cohort studies were categorized as low risk of bias as shown by Newcastle-Ottawa Scale qualifier, the certain of evidence was low or very low. Since all included studies had a non-randomized design, the certainty of the evidence was already expected to be low. However, primary studies presented some methodological issues that downgraded the certainty of the evidence. The main concern was inconsistency in all meta-analyses was high due to a high methodological heterogeneity among studies. Some studies included failed to report a clear sample size calculation. Despite having 9 available trials, the sample size of the studies was low, and all studies are prospective non-randomized trials, thus lacking blinding and randomization of the selected sample. Most of the reviewed studies do not provide information on potential confounding factors. Identifying these factors should be noted to minimize potential differences between evaluations, as they may influence the outcome direction. This also applies to different protocols in electromyographic assessment and differences in the amplitude and average standard deviation of electromyographic potentials in some studies. Large variations affect the homogeneity of variance between groups, compromising the comparison between them, and hindering the conduct of a more robust meta-analysis.

Overall, the present systematic review results indicate that rapid maxillary expansion is a safe, effective, and important treatment for unilateral posterior crossbite, showing positive effects on activity muscles Nonetheless, the quality of evidence on this topic needs improvement. More studies should be conducted, based on rigorous methodological standards, preferably through randomized controlled trials. Additionally, sample size calculation should be performed to establish adequate statistical power. The selection of patients with unilateral crossbite should be made using well-defined and calibrated diagnostic criteria among examiners. The same applies to data analysis, which should be conducted validly and reliably. Reproducibility of measurements and electromyographic parameters should be as uniform as possible, with consolidation of protocols to be adopted. Likewise, potential confounding factors should be evaluated and identified to minimize any influence on the evaluated outcomes. In addition, longer follow-ups are needed to assess the long-term effects of treatment and further elucidate the findings and evidence available in this systematic review.

## Conclusions

The findings indicated that utilizing rapid maxillary expansion for the correction of dental unilateral posterior crossbite serves as a significant therapeutic intervention. In addition to addressing bone and dental irregularities, it enhances the function of masticatory muscles, specifically the masseter and temporal muscles.

## Figures and Tables

**Table 1:** General characteristics of included studies.

Authors, Year (local)	Design Study	Sample Size (n) and Sex (M/F)	Mean Age at Start of Treatment in Years	Mean Duration of Therapy	Outcome Variable	Outcomes Evaluated	Follow-up	Outcomes		Conclusion
								Masseter Muscle Activity	Temporal Muscle Activity	
Spolaor et al., 2020 (Padova, Italy)	Prospective	15 (M/F = NR)	9 ± 2.28	Hyrax appliance Between 4 and 6 weeks of expansion 6 months of use (contention)	EMG	Masseter and temporal. Activities in mastication	3 months	Percentage ± SD unilateral posterior crossbite on the right (UPCBr) CHEWING TASK Masseter right / Left Baseline: 78 ± 20 / 76 ± 21 Post-treatment: 75 ± 26 / 81 ± 20 Post-Follow-up: 75 ± 30 / 81 ± 23 unilateral posterior crossbite on the left (UPCBl) Masseter right / Left Baseline: 82 ± 22 / 78 ± 27 Post-treatment: 63 ± 38 / 62 ± 32 Post-Follow-up: 71 ± 30 / 79 ± 30	Percentage ± SD unilateral posterior crossbite on the right (UPCBr) CHEWING TASK Temporal right / Left Baseline: 75 ± 25 / 71 ± 23 Post-treatment: 72 ± 29 / 59 ± 30 Post-Follow-up: 81 ± 24 / 73 ± 29.5 unilateral posterior crossbite on the left (UPCBl) Temporal right / Left Baseline: 78 ± 23 / 71 ± 28 Post-treatment: 72 ± 26 / 70 ± 36 Post-Follow-up: 77 ± 20 / 82 ± 28	Surface electromyography demonstrated a relationship between the correction of a maxillary transverse discrepancy and the restoration of a muscle's activation patterns comparable to healthy subjects for both Temporal and Masseter.
Michelotti et al., 2019 (Naples, Italy)	Prospective	29 (M: 13; F:16)	9.6 ± 1.6	Two-band palatal expander 10 to 16 days of expansion 6 months of use (contention)	EMG	Masseter and Anterior Temporalis. Activities during maximum voluntary Clenching and mastication	6 months	Percentage of overlapping coefficient ± SD: Masseter CLENCH Baseline: 83.0 ± 7.4 Post-treatment: 82.4 ± 10.2 Post-Follow-up: 84.9 ± 5.6 P value: 0.311	Percentage of overlapping coefficient ± SD: Anterior temporalis CLENCH Baseline: 84.7 ± 5.9 Post-treatment: 83.2 ± 5.5 Post-Follow-up: 83.6 ± 10 P value: 0.666	UPCB does not contribute to an asymmetric activation of AT anterior temporalis and masseter during functional tasks. The treatment of UPCB by RME did not determine a more symmetric activity of the assessed muscles.
Pimentel et al., 2019 (Ribeirão Preto, Brazil)	Prospective	20 (M: 7; F: 13)	9.0 ± 3.0	Haas expander Until overcorrection of the posterior crossbite was achieved	EMG	Masseter and Anterior temporalis. Cross side and not crossed side on Electromyographic activity and temporomandibular variables.	3 months	Mean values (µV Cross / Not crossed / Difference Baseline – Post treatment Rest (uV) 2.45 / 2.43 / 0.23 - 2.978 / 2.974 / 0.04 Functional CLENCH 92.19/ 86.54/ 5.65 - 82.95/ 82.94 Long CLENCH 169.6 / 169.1 / 0.5 - 155.6 / 155.3 / 0.3 Chewing – Cross side 8.6 / 14.5 / 5.8 - 15.1 / 16.6 / 1.4 Chewing – Not crossed side 15.12 / 8.59 / 6.52 - 15.91 / 14.73 / 1.18 Habitual chewing (Hz) 11.0 / 10.9 / 0.1 - 13.9 / 13.2 / 0.7	Mean values (µV Cross / Not crossed / Difference Baseline – Post treatment Rest (uV) 2.45 / 2.43 / 0.23 - 3.33 / 3.39 / 0.06 Functional CLENCH 104.8 / 97.16 / 7.64 – 104 / 100.5 / -3.5 Long CLENCH 175 / 167.6 / 7.4 - 167.1 / 172.3 / 5.2 Chewing – Cross side 12.8 / 17.4 / 4.53 - 16.3 / 17.4/ 1.07 Chewing – Not crossed side 17.6 / 13.0 / 4.6 - 17.7 / 16.3 / -1.4 Habitual chewing (Hz) 13.1 / 14.1 / 0.9 - 14.7 / 14.1 / 0.6	The proposed treatment did not lead to the occurrence of joint noises and improved the functional pattern of electromyographic activity during chewing at the end of treatment.
Di Palma. et al., 2017 (L´Aquila, Italy)	Prospective	21 (M: 10; F:11)	9.8 ± 1.6	Hyrax appliance Until overcorrection of the posterior crossbite was achieved	EMG	Masseter and Anterior temporalis. Activities in: neuromuscular equilibrium In maximum Clench	3 months	Percentage of overlapping coefficient ± SD: Masseter CLENCH Baseline: 84.06 ± 8.43 Post-Follow-up: 85.64 ± 5.63 P value: 0.543	Percentage of overlapping ± SD: Anterior temporalis CLENCH Baseline: 86.57 ± 3.86 Post: 85.65 ± 5.62 P value: 0.308	In children without pre-treatment EMG alterations, no variations in standardized muscular activity after RME were found. The treatment did not alter the equilibrium of the masseter and temporal muscles.
Galbiati et al., 2016 (Milan, Italy)	Prospective	71 (M: 36; F:35)	Range: 6 to 10 years	Hyrax appliance until overcorrection of the posterior crossbite was achieved	EMG	Masseter (Right and Left) and Anterior temporal (Right and Left) activities in: AMR, AMR TENS, COTTON and CLENCH	6 months	Mean values (µV) ± SD Masseter: Right / Left AMR Baseline: 2.3 ± 0.7 / 2.3 ± 0.4 Post-Treatment: 2.4 ± 1.4 / 3.4 ±1.2 P value: 0.55 / 0.02 AMR TENS Baseline: 1.7 ± 0.4 / 1.7 ± 0.8 Post-Treatment: 2.2 ± 1.1 / 2.7 ± 1.3 P value: 0.04 / 0.03 COTTON Baseline: 55.5 ± 17.8 / 46.4 ± 28.4 Post-Treatment: 48.2 ± 21.7 / 53.3 ± 14.8 P value: 0.73 / 0.57 CLENCH Baseline: 66.8 ± 21.5 / 48.8 ± 16.1 Post-Treatment: 47.7 ± 11.4 / 54.9 ± 12.9 P value: 0.03 / 0.48	Mean values (µV) ± SD Anterior Temporal: Right / Left: AMR Baseline: 2.7 ± 0.7 / 3.7 ± 0.4 Post: 4.2 ± 2.3 / 3.2 ± 1.3 P value: 0.04 / 0.28 AMR TENS Baseline: 2.4 ± 0.2 / 3.2 ± 0.4 Post-Treatment: 3.3 ± 1.4 / 3.7 ± 1.8 P value: 0.28 / 0.29 COTTON Baseline: 58.2 ± 48.9 / 64.7 ± 41.1 Post-Treatment: 56.1 ± 31.2 / 65.3 ± 25.4 P value: 0.95 / 0.85 CLENCH Baseline: 77.3 ± 17.8 / 76.7 ± 19.7 Post-Treatment: 55.1 ± 11.4 / 63.3 ± 17.3 P value: 0.01 / 0.48	In this study, the muscular activity was increased after therapy producing important changes in muscular tone.
Maspero et al., 2015 (Milan, Italy)	Prospective	55 (M: 27; F:28)	6 to 10 years old	Hyrax appliance until overcorrection of the posterior crossbite was achieved	EMG	Masseter (Right and Left) and Anterior temporal (Right and Left) activities in: AMR, AMR TENS, COTTON and CLENCH	6 months	Mean values (µV) ± SD Masseter: Right / Left AMR Baseline: 2.1± 0.8 / 2.1± 0.6 Post-Treatment: 2.4 ± 1.5 / 3.2±1.7 P value: 0.66 / 0.04 AMR TENS Baseline: 1.4 ± 0.4 / 1.6 ± 0.3 Post-Treatment: 2.1 ± 1.1 / 2.8 ± 1.6 P value: 0.05 / 0.05 COTTON Baseline: 55.4 ± 46.8 / 46.1 ± 28.8 Post-Treatment: 48.4 ± 27.7 / 53.4 ± 14.5 P value: 0.37 / 0.26 CLENCH Baseline: 66.1 ± 45.5 / 48.3 ± 26.7 Post-Treatment: 47.4 ± 40.6 / 54.7 ± 32.9 P value: 0.04 / 0.46	Mean values (µV) ± SD Anterior Temporal: Right / Left: AMR Baseline: 2.4 ± 0.9 / 3.3 ± 0.5 Post-Treatment: 4.4 ± 2.4 / 3.3 ± 1.1 P value: 0.03 / 0.91 AMR TENS Baseline: 2.2 ± 0.6 / 3.0 ± 0.5 Post-Treatment: 3.1 ± 1.3 / 3.8 ± 1.5 P value: 0.2 / 0.2 COTTON Baseline: 58.2 ± 48.9 / 64.7 ± 41.1 Post-Treatment: 56.1 ± 31.2 / 65.3 ± 25.4 P value: 0.86 / 0.92 CLENCH Baseline: 75.9 ± 47.4 / 76.0 ± 49.9 Post-Treatment: 53.3 ± 41.9 / 63.1 ± 37.2 P value: 0.04 / 0.29	Electromyographic analysis showed that activity of the masseter and temporalis muscles increased significantly after the expansion appliance was removed during rest, dental Clenching and habitual chewing. Rapid palatal expansion produces important changes in the muscular tone and it increases the muscular activity of the masticatory muscles.
Martín et al., 2012 (Madrid, Spain)	Prospective	25 (M: 10; F: 15)	Mean age: 12.5	Quad-helix (QH) appliance Until overcorrection of the posterior crossbite was achieved	EMG	Masseter and Anterior, Posterior temporal activities in: Chewing task, Clenching, Rest Position and Swallowing	12 months	Mean values (µV) ± SD Masseter CHEWING TASK Baseline: 35.72 ± 19.48 Post-treatment: 55.69 ± 23.26 Post-Follow-up: 57.67 ± 20.73 P value: <0.001 CLENCH Baseline: 202.9 ± 92.17 Post-treatment: 295.10 ± 84.66 Post-Follow-up: 328.81 ± 86.91 P value: <0.001 AMR Baseline: 1.66 ± 1.24 Post-treatment: 0.93 ± 0.54 Post-Follow-up: 1.10 ± 0.67 P value: <0.001 SWALLOWING Baseline: 53.68 ± 40.50 Post-treatment: 64.20 ± 43.26 Post-Follow-up: 62.15 ± 41.27 P value: 0.283	Mean values (µV) ± SD Temporalis: Anterior / Posterior CHEWING TASK Baseline: 44.81 ± 19.38 / 21.47 ± 14.73 Post-treatment: 59.8 ± 21.9 / 36.4 ± 18.4 Post-Follow-up: 62.3 ± 24.1 / 37.0 ± 17.6 P value: 0.006 / <0.001 AMR Baseline: 3.27 ± 2.70 / 3.27 ± 2.77 Post-treatment: 1.66 ± 1.13 / 3.38 ± 2.60 Post-Follow-up: 1.48 ± 0.82 / 4.07 ± 2.29 P value: <0.001 / 0.099 SWALLOWING Baseline: 61.18 ± 60.64 / 61.56 ± 51.00 Post-treatment: 53.5 ± 54.2 / 92.3 ± 70.0 Post-Follow-up: 58.1 ± 53.8 / 57.8 ± 33.7 P value: 0.122 / 0.058 Anterior Temporalis CLENCH Baseline: 245.36 ± 63.36 Post-treatment: 317.79 ± 69.27 Post-Follow-up: 327.93 ± 87.41 P value: <0.001	During mastication, MA activity increased significantly and its asymmetry was corrected post-treatment. During Clenching, cross-bite side AT and MA activity increased significantly posttreatment and remained stable after retention, and MA/AT ratio reversed.
De Rossi. et al., 2009 (Ribeirão Preto, Brazil)	Prospective	27 (M: 12; F: 15)	Average age: 8.5 years	Bonded acrylic-splint appliance until overcorrection of the posterior crossbite was achieved	EMG	Masseter and temporal activities in: Rest Position, Dental Clenching, Habitual Chewing	5 months (range, 4.1-6.2 months)	Mean values (µV) ± SD Masseter: Right / Left AMR Baseline: 7.28 ± 4.97 / 7.00 ± 3.53 Post-Treatment: 11.58 ± 12.15 / 12.32 ± 11.72 P value: 0.025 / 0.013 CLENCH Baseline: 113.17 ± 26.41 / 112.51 ± 34.36 Post-Treatment: 143.65 ± 116.38 / 149.06 ± 99.15 P value: 0.197 / 0.087 CHEWING TASK Baseline: 56.80 ± 29.87 / 65.54 ± 27.96 Post-Treatment: 96.28 ± 128.22 / 95.16 ± 93.60 P value: 0.116 / 0.099	Mean values (µV) ± SD Anterior Temporal: Right / Left: AMR Baseline: 7.0 ± 4.09 / 8.5 ± 4.9 Post-Treatment: 12.0 ± 11.2 / 12.7 ±10.1 P value: 0.008 / 0.013 CLENCH Baseline: 102.06. ± 28.5/ 102.14 ± 26.98 Post-Treatment: 116.6 ± 49.2 / 135.6 ± 65.6 P value: 0.189 / 0.009 CHEWING TASK Baseline: 50.29 ± 22.98 / 58.02 ± 23.92 Post-Treatment: 66.5 ± 32.3 / 81.3 ± 43.4 P value: 0.026 / 0.007	Electromyographic analysis showed that activity of the masseter and temporalis muscles increased significantly after the expansion appliance was removed during rest, dental Clenching, and habitual chewing.
Kecik et al., 2007 (Ankara, Turkey)	Prospective	35 (M: 15; F: 20)	UPCB mean age of 10.6 ± 1.4 years	Quad-helix appliance 3 months of expansion	EMG	Masseter and Anterior temporal activities in: Rest Position, swallowing and Clenching	3 months	Mean values (µV) ± SD Masseter AMR Baseline: −1.73 ± 0.27 Post-Follow-up: 0.20 ± 0.27 P value: <0.01 SWALLOWING Baseline: −1.73 ± 0.27 Post-Follow-up: 0.20 ± 0.27 P value: <0.01 CLENCH Baseline: 3.25 ± 1.37 Post-Follow-up: 0.63 ± 0.03 P value: <0.001	Mean values (µV) ± SD Anterior Temporalis AMR Baseline: −2.09 ± 1.28 Post-Follow-up: −0.27 ± 0.53 P value: <0.001. SWALLOWING Baseline: −2.09 ± 1.28 Post-Follow-up: −0.27 ± 0.53 P value: <0.001 CLENCH Baseline: −2.97 ± 0.43 Post-Follow-up: −0.06 ± 0.02 P value: <0.001	RME recordings of the masseter and anterior temporalis muscles during rest, swallowing, and Clenching showed differences between both periods. Unbalanced masticatory muscle activity improves with the elimination of the mandibular shift.

EMG: Electromyography; UPCB: Unilateral posterior crossbite; AMR – muscular activity at rest position; AMR TENS: muscular activity at rest after transcutaneal electrical neural stimulation; COTTON – force exerted on the maximum voluntary Clenching on cotton rolls; CLENCH – force exerted on the maximum voluntary Clenching on teeth

**Table 2:** Risk bias of the selected studies.

Studies	Selection				Comparability		Outcome			Total
	Exposed Cohort^*^	Non exposed cohort^*^	Ascertainment of exposure	Outcome of interest not present at start	Main Factor	Additional Factor	Assessment of outcome	Follow-up long enough	Adequacy of follow-up	
Spolaor et al. (39)	⭒	⭒	⭒	⭒	⭒	⭒	⭒	⭒	⭒	9
Michelotti et al. (43)	⭒	⭒	⭒	⭒	⭒	0	⭒	⭒	⭒	8
Pimentel et al. (44)	⭒	⭒	⭒	⭒	⭒	0	⭒	⭒	⭒	8
Di Palma et al. (42)	⭒	0	⭒	⭒	⭒	0	⭒	⭒	⭒	7
Galbiati et al. (41)	⭒	0	⭒	⭒	⭒	0	⭒	⭒	⭒	7
Maspero et al. (18)	⭒	0	⭒	⭒	⭒	⭒	⭒	⭒	⭒	8
Martín et al. (40)	⭒	0	⭒	⭒	⭒	0	⭒	⭒	⭒	7
De Rossi et al. (20)	⭒	0	⭒	⭒	⭒	⭒	⭒	⭒	⭒	7
Kecik et al. (38)	⭒	⭒	⭒	⭒	⭒	⭒	⭒	⭒	⭒	9

**Table 3:** Meta-analysis results in each type of temporal and masseter muscle activity. Certainty of the evidence of each meta-analysis.

Variables	References	n	Meta-analysis results					Certainty of the evidence
Masseter Muscle			MD	95%-CI	Meta-analysis p-value	Heterogeneity I^2^ (p-value)	Funnel plot bias
CLENCH	Di Palma et al. 2017 Michelotti et al. 2019	50	1.7791	-0.8853; 4.4436	0.1906	0% (0.9091)	No	⨁◯◯◯ VERY LOW
CLENCH (Right side)	De Rossi et al. 2009 Maspero et al. 2015 Galbiati et al. 2016	153	-18.3612	-23.7222; -13.0003	<0.0001	56% (0.1008)	Yes	⨁◯◯◯ VERY LOW
CLENCH (Left side)	De Rossi et al. 2009 Maspero et al. 2015 Galbiati et al. 2016	153	6.5195	2.1358; 10.9032	0.0036	10.8% (0.3261)	No	⨁⨁◯◯ LOW
AMR	Kecik et al. 2009 Martín et al. 2012	60	1.8060	1.6827; 1.9293	<0.0001	98.7% (<0.0001)	Yes	⨁◯◯◯ VERY LOW
AMR (Right side)	De Rossi et al. 2009 Maspero et al. 2015 Galbiati et al. 2016	153	0.1927	-0.0897; 0.4751	0.1811	35.7% (0.2110)	No	⨁⨁◯◯ LOW
AMR (Left side)	De Rossi et al. 2009 Maspero et al. 2015 Galbiati et al. 2016	153	1.1124	0.8624; 1.3623	<0.0001	37.5% (0.2019)	No	⨁⨁◯◯ LOW
AMRTENS (Right side)	Maspero et al. 2015 Galbiati et al. 2016	126	0.5873	0.3829; 0.7917	<0.0001	0% (0.3415)	No	⨁⨁◯◯ LOW
AMRTENS (Left side)	Maspero et al. 2015 Galbiati et al. 2016	126	1.0810	0.8072; 1.3549	<0.0001	0% (0.4822)	No	⨁⨁◯◯ LOW
COTTON (Right side)	Maspero et al. 2015 Galbiati et al. 2016	126	-7.2487	-13.1926; -1.3047	0.0168	0% (0.9003)	No	⨁⨁◯◯ LOW
COTTON (Left side)	Maspero et al. 2015 Galbiati et al. 2016	126	7.0733	1.4648; 12.6817	0.0134	0% (0.9448)	No	⨁⨁◯◯ LOW
SWA	Kecik et al. 2009 Martín et al. 2012	60	1.9302	1.8037; 2.0567	<0.0001	0% (0.5717)	No	⨁◯◯◯ VERY LOW
Temporal Muscle								
CLENCH	Di Palma et al. 2017 Michelotti et al. 2019	50	-0.9781	-3.3781; 1.4220	0.4245	0% (0.9452)	No	⨁◯◯◯ VERY LOW
CLENCH (Right side)	De Rossi et al. 2009 Maspero et al. 2015 Galbiati et al. 2016	153	-20.5402	-25.1475; -15.9330	<0.0001	81.4% (0.0046)	Yes	⨁◯◯◯ VERY LOW
CLENCH (Left side)	De Rossi et al. 2009 Maspero et al. 2015 Galbiati et al. 2016	153	-11.2967	-16.8887; -5.7047	<0.0001	82.2% (0.0036)	Yes	⨁◯◯◯ VERY LOW
AMR (Right side)	De Rossi et al. 2009 Maspero et al. 2015 Galbiati et al. 2016	153	1.7324	1.3031; 2.1617	<0.0001	38.6% (0.1960)	No	⨁⨁◯◯ LOW
AMR (Left side)	De Rossi et al. 2009 Maspero et al. 2015 Galbiati et al. 2016	153	-0.2399	-0.4643; -0.0155	0.0362	77.8% (0.0112)	Yes	⨁◯◯◯ VERY LOW
AMRTENS (Right side)	Maspero et al. 2015 Galbiati et al. 2016	126	0.9000	0.6517; 1.1483	<0.0001	0% (1.000)	No	⨁⨁◯◯ LOW
AMRTENS (Left side)	Maspero et al. 2015 Galbiati et al. 2016	126	0.6539	0.3546; 0.9532	<0.0001	0% (0.3261)	No	⨁⨁◯◯ LOW
COTTON (Right side)	Maspero et al. 2015 Galbiati et al. 2016	126	-2.1000	-12.2282; 8.0282	0.6845	0% (1.000)	No	⨁⨁◯◯ LOW
COTTON (Left side)	Maspero et al. 2015 Galbiati et al. 2016	126	0.6000	-7.8362; 9.0362	0.8891	0% (1.000)	No	⨁⨁◯◯ LOW

## Data Availability

All the data generated or analyzed during this study are included in this published article and its supplementary information files.
